# The Aging Vasculature: Glucose Tolerance, Hypoglycemia and the Role of the Serum Response Factor

**DOI:** 10.3390/jcdd8050058

**Published:** 2021-05-17

**Authors:** Hazel Aberdeen, Kaela Battles, Ariana Taylor, Jeranae Garner-Donald, Ana Davis-Wilson, Bryan T. Rogers, Candice Cavalier, Emmanuel D. Williams

**Affiliations:** 1 Department of Biomedical Sciences, Baptist Health Sciences University, Memphis, TN 38103, USA; hazel.aberdeen@bchs.edu or; 2 Department of Biology and Chemistry, Southern University and A&M College, Baton Rouge, LA 70813, USA; kaela_battles_00@subr.edu (K.B.); ariana_taylor_00@subr.edu (A.T.); jeranae_garner-don00@subr.edu (J.G.-D.); ana.davis@sus.edu (A.D.-W.); Bryan_Rogers@subr.edu (B.T.R.); candice_cavalier@subr.edu (C.C.)

**Keywords:** aging, serum response factor, vascular, glucose, hypoglycemia, heart

## Abstract

The fastest growing demographic in the U.S. at the present time is those aged 65 years and older. Accompanying advancing age are a myriad of physiological changes in which reserve capacity is diminished and homeostatic control attenuates. One facet of homeostatic control lost with advancing age is glucose tolerance. Nowhere is this more accentuated than in the high proportion of older Americans who are diabetic. Coupled with advancing age, diabetes predisposes affected subjects to the onset and progression of cardiovascular disease (CVD). In the treatment of type 2 diabetes, hypoglycemic episodes are a frequent clinical manifestation, which often result in more severe pathological outcomes compared to those observed in cases of insulin resistance, including premature appearance of biomarkers of senescence. Unfortunately, molecular mechanisms of hypoglycemia remain unclear and the subject of much debate. In this review, the molecular basis of the aging vasculature (endothelium) and how glycemic flux drives the appearance of cardiovascular lesions and injury are discussed. Further, we review the potential role of the serum response factor (SRF) in driving glycemic flux-related cellular signaling through its association with various proteins.

## 1. Introduction

The exponential expansion of the older adult demographic (65 and older) in the United States is unique in the nation’s history. By the midpoint of the twenty-first century, it is estimated that Americans aged 65 or older will number approximately 90 million people, and the number of older Americans just merely a decade into the twenty-first century will nearly double [1].

The rapid aging of the United States population is influenced by two primary factors: (1) Americans are living longer than in previous decades and (2) there are more older adults aged 65 and older than in previous generations, post-World War II. Americans are now living beyond their 70s, with many achieving centenarian status. The oldest of the “baby boomers” reached age 65 in 2011, setting off a phenomenon in the United States not previously observed. Since the start of 2011, and every day for the next 15 to 20 years, approximately 8000–10,000 Americans will observe their 65th birthday. In the year 2030, when the last baby boomer turns 65, the demographic makeup of our country will have changed greatly. Moreover, it is estimated that twenty percent of all Americans—approximately 77 million people—will be aged 65 years or older [2].

The aging demographic has had a widespread impact on essentially every component of American society. At each transition from one life stage to the next in the lifespan of those born after World War II, the United States has been impacted by their increased numbers and needs—from robust demand in commercial adolescent products as they entered into their teen years during the late 1940s and 1950s, to the infrastructural impacts via construction of thousands of new schools during the following decades, to the housing boom of the 1970s. Modern times demonstrate that the baby boomer’s population continues to exercise its influence. Unquestionably, this occurrence will have its most significant impact on United States public health care systems. Public health plays an essential role in supporting those in dire need, bridging patients and communities to available resources, and equally important, promoting healthy aging because of its impact on environmental factors. The public health sector is readily well-positioned to meet the growing needs and demands of a rapidly aging nation [2,3,4].

During the last half of the twentieth century, robust health care initiatives and rapid advances in therapeutic treatment led in part to an increase in average life expectancy in the United States. This nearly 30 year gain in life expectancy within a hundred-year span had never been observed. Many of the pathologies that claimed our inhabitants of previous generations, including polio and typhoid, cease to be the “doom and gloom” diagnosis that they once were at the turn of the century. While these “threats of old” routinely present significant health challenges in the United States, these manifestations are no longer the morbidity and mortality threat in adults they once were. However, other pathologies have continued to be staples in the leading causes of death among Americans. Since 1909, heart disease has been the leading cause of death among American adults every year except in the period 1918–1920, when the Spanish Flu epidemic occurred [4]. Moreover, since 1935, cancer has been the next leading cause of mortality in Americans [5].

**Figure 1:**
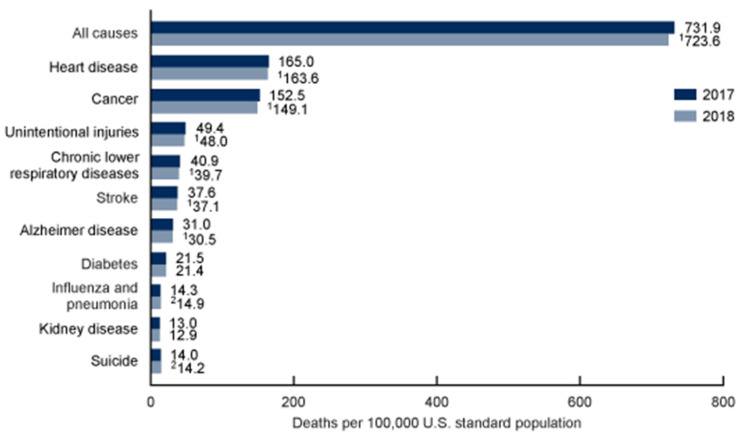
The 2018 Center for Disease Control and Prevention report on the leading causes of death from 2017 to 2018. ^1^ Statistically significant decrease in age-adjusted death rate from 2017 to 2018 (*p* < 0.05).^2^ Statistically significant increase in age-adjusted death rate from 2017 to 2018 (*p* < 0.05).NOTES: A total of 2,839,205 resident deaths were registered in the United States in 2018. The 10 leading causes of death accounted for 73.8% of all deaths in the United States in 2018. Causes of death are ranked according to number of deaths. Rankings for 2018 were the same as in 2017. Data table for Figure 2 includes the number of deaths for leading causes.

According to the 2018 Centers for Disease Control & Prevention, both cancer and heart disease risks are increased with advancing age (Figure 1; [6]).

### 1.1. The Aging Heart

**Figure 2:**
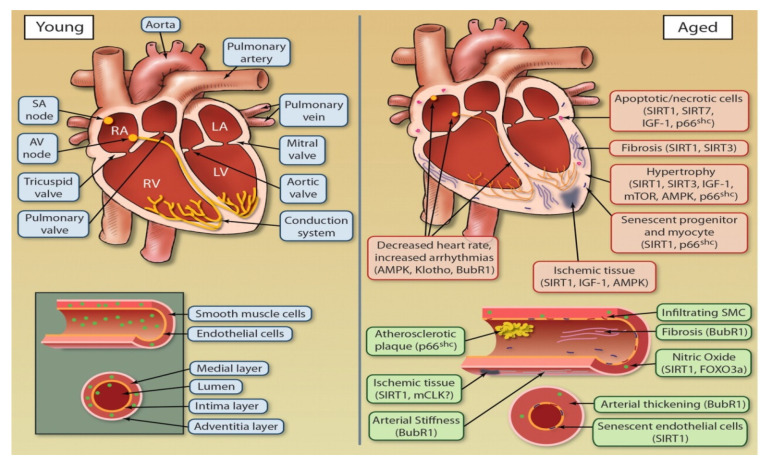
Comparative model of the aging heart and vasculature in young vs. older populations.

Aging is highlighted with a gradual decline in reserve capacity as well as physiological processes that lead to a greater risk of pathological conditions. By pumping oxygen-rich blood throughout the body, the fitness of the cardiovascular system is pivotal for the other organ systems and overall fitness and health of the organism. Advancing age has a significant effect on the heart and vasculature, ultimately leading to a gradual predisposition in cardiovascular episodes including atherosclerosis and stroke [7]. Previous studies demonstrate aged cardiovascular tissues are highlighted by pathological changes that include, hypertrophy, arterial stiffness, and endothelial cell dysfunction (Figure 2; [8]). 

The heart also undergoes significant changes with advancing age that affect cellular dynamics including a reduced number of cardiomyocytes as a result of increased apoptosis and necrosis. In addition, advancing aging leads to a reduction in the replenishment of cardiomyocytes from stem cell reserves [9,10]. Advancing age further renders cardiomyocytes sensitive and vulnerable to various stress, including oxidative stress. Elevated reactive oxygen species (ROS) production with age results in reduced cardiomyocyte populations [11,12]. Necrotic episodes in cardiomyocytes results in the paracrine release of contents into the surroundings; this phenomenon greatly affects survival of neighboring cardiomyocytes, in addition to promoting the development and maintenance of pro-inflammatory and fibrotic environments in the heart [13].

Cardiomyocytes are very much active in cell division. Recent studies have elucidated that cardiac regeneration is an essential physiological process for propagation of vascular function [14]. The regeneration rate of cardiomyocyte populations due to advancing age may not be adequate to sustain cell population due to cardiomyocyte loss. Mitotic cardiac tissue contains a small population of cardiac stem cells in addition to a limited pool of incompletely differentiated cardiomyocytes that can re-enter the cell cycle from quiescence [15,16].

The overall health of the vasculature and cardiac systems are not entirely separate of each other; each system significantly impacts the other. For example, an augmentation in arterial stiffness leads to concomitant changes in the heart wall (the myocardium). These changes encompass left ventricular hypertrophy and fibroblast cell division; these cardiovascular events have been implicated in decreased cardiac function and increased fibrotic scar deposition [17]. Heart rate is also affected by advancing age. Both maximum heart rate and rhythm are decreased with advancing age [18]. Heart rate is also influenced by the reduced number of cells in the sinoatrial cavity in addition to other physical changes in the heart, including fibrotic deposition, which slow rate of electric signaling within the heart [19]. 

After the age of 30, the end diastolic volume rate in the left ventricles begins to attenuate. This decline is countered by increasing arterial contraction to maintain stroke volume, sustaining ejection fraction [20]. With advancing age, however, the LV ejection fraction, rhythmic control of heart rate by the sympathetic nervous system, and sensitivity to β-adrenergic receptor activity decline. This age-associated decrease in cardiac output causes cardiac hypertrophy (increased cardiac muscle mass [21]). Hypertrophy of the ventricles is the by-product of a size increase in individual cardiomyocytes and due to both reversible stimuli (i.e., exercise) or irreversible pathological occurrences [22,23].

### 1.2. The Aging Vasculature

The physical stress, fatigue, and frailty observed in the elderly can, in large part, be in large part attributed to vascular aging. Although age-associated changes in vascular function are hallmarks for the onset of cardiovascular disease, interventions on cardiac function can attenuate or rapidly accelerate the onset of the disease. It is therefore imperative to understand the role of advancing age and other pathophysiological (both age-dependent and independent) manifestations that affect the aging vasculature.

The effects of advancing age on the vasculature result in both arterial thickening and stiffness and a compromised endothelium. From a clinical standpoint, these age-associated changes result in increased systolic pressure, which in turn, serves as a major risk factor and gateway for development of atherosclerosis and coronary artery disease [24]. Further dysfunction of the vasculature associated with aging contributed to the progression and severity of many pathologies, including loss of adequate supply of oxygen to tissues (ischemia), vascular constriction (hypertension), or macular degeneration. The vasculature undergoes structural and functional changes with age that include enlargement of the vascular lumen with concomitant arterial wall thickening and an increase in dysfunction of the endothelium; the latter negatively impacts vasodilation and promotes vascular stiffness [25].

### 1.3. The Aging Vasculature: Endothelium

The vascular endothelium is the immediate point of contact between the blood and tissue and is more than a simple semipermeable barrier. It has anti-coagulatory properties and is responsible for producing biological factors that in part regulate vascular tone and homeostasis [26]. The endothelium consists of approximately 1–9 × 10^13^ endothelial cells forming an organ over 500 g and is thought to arise from the mesoderm [27]. Endothelial cells form the lining of blood vessels and provides a barrier between the vessel wall and blood. In addition to this role, endothelial cells fulfil multiple functions with both metabolic and synthetic functions. Endothelial cells respond to physical and chemical factors within circulation and in response regulates a plethora of metabolic functions, including hemostasis and inflammation. Vascular angiogenesis, or de novo synthesis of blood vessels, is another role endothelial cells play a large role in and endothelial cells also have a pivotal role in angiogenesis and vasculogenesis. Many disease states, including cancer, involve structural and physiological changes to the endothelium [28].

Early reversible signs of atherosclerosis (i.e., fatty streaks and lesions) are already present in utero [29,30] and frequently augment to full manifestation in early to mid-twenties [31]. This slow-progressing phenomenon ends in clinical episodes, including ischemia and cardiac arrest. While atherosclerosis is a vascular event that gradually manifests, vascular aging and atherosclerosis are not mutually exclusive [32].

Endothelial cell dysfunction also accompanies advancing age [33,34,35,36,37]. With advancing age, endothelial cells lose their ability to divide after tissue injury [30]. In addition endothelial cells that form a protective barrier are less cohesive and porous. This permits vascular smooth muscle cells (VSMCs) to circulate and induce intimal thickening [38]. Nitric oxide (NO) is referred to as the “master” endothelial-derived autacoid [39] and anti-atherosclerotic compound [40]. In addition to its properties as a vasodilator, NO has far-reaching properties that include effects on leukocyte adhesion [41], platelet aggregation, and vascular smooth muscle cell (VSMCs) proliferation [42]. Further, while NO functions as an antioxidant and ceases lipid peroxidation, it itself acts as a direct oxidant by the modulating the rapid formation of peroxynitrite from reactions with the various superoxide anions [43].

Multiple reports suggests endothelial NO synthase (eNOS) expression and NO production decline with age [44,45,46], whereas other authors contradict these findings, with data suggesting eNOS expression to be increased with advancing age [47,48]. Increased vascular formation of O_2_^−^ has been reported; O_2_^−^ is a known to facilitate endothelial-induced dilation since scavenging of radicals improves vascular endothelium-dependent sensitivity. An increase in O_2_^−^ and NO production leads to the uncoupling of the eNOS through production of the peroxynitrite species [49]. Not surprisingly, the cellular antioxidative defense system is weakened with advancing age [50]. The extracellular concentration of hydrogen peroxide-forming superoxide dismutase (SOD), but not the intracellular SOD content in rats decreases. While the biological importance of this phenomena remains unclear, the altered extracellular SOD activity is most likely, in part, a result of the reduced NO bioavailability. The age-associated increase in peroxynitrite appears to negatively affect antioxidative enzymes, especially the manganese SOD (MnSOD) in mitochondria [50].

Numerous other mechanisms can facilitate eNOS activity. Hemodynamic shear stress, the frictional force exerted by blood flow on endothelial cell surface, is one of the most potent inducers of eNOS activity [51,52]. As vessels age, the amount of hemodynamic stress is reduced, in part, to weakened blood flow caused by the decline in left ventricular heart function. Consequently, endothelial cells become less sensitive to shear stress resulting in a decline in the protective benefits of nitric oxide. NO synthase and its essential cofactor tetrabiopterin (BH_4_) are also affected with advancing age and also mediate vascular function by indirect association with NO. Deficiency of tetrahydrobiopterin (BH_4_), declines with advancing age, partially due to oxidation by free radicals [53]. As a result, NO synthase is no longer able to synthesize NO from arginine, a process known as nitric oxide uncoupling.

### 1.4. Endothelial Cellular Senescence

Most mammalian cells, including cardiomyocytes, have a limited replicative capacity. These cells withdraw from the cell cycle and enter a state of permanent growth arrest, termed senescence [54]. Senescent cells are very metabolically active, but physically altered via the cytoskeleton. These cells express biomarkers of growth arrest including senescence-associated enzymes such as the acidic β-galactosidase (SA-β-gal) [55]. Under ideal physiological growth parameters, endothelial cells proliferate and exhibit a turnover rate of approximately three years. Several processes, most notably tissue injury, stimulate endothelial cell proliferation. As a result, the attenuated wound healing and angiogenic processes typically observed in the older adults have been partly attributed to endothelial cell senescence [56]. However, whether endothelial cell senescence is an aging-associated or a vascular disease-associated process remains a topic of research debate. For example, an increase in SA-β-gal activity has been observed in endothelial cells within atherosclerotic lesions, but not in coronary artery models that are void of signs of atherosclerosis [57]. Another biomarker of senescence is telomere length. Telomeres are crucial for chromosomal stability and with each DNA duplication, approximately 150 base pairs of telomeric DNA are not copied. Senescence is only reached when telomeres are shortened below a basal threshold [58]. Interestingly, telomere length is inversely correlated to age in in vivo endothelial cell models; with increased age, telomere length decreases [59]. 

Telomere attrition is opposed by the enzyme telomerase reverse transcriptase (TERT). TERT is not expressed in most somatic cells but is robust in germ line cells and most tumor cells, limiting senescence in these cell lines. In vitro endothelial cells, however, have been reported to possess TERT activity, which may in part explain delays the onset of senescence in these models [60]. In vitro aging leads to a reduction in NO formation, which in consequence results in a loss of TERT enzymatic activity. Previous studies have shown that treatment with exogenous nitric oxide can restore TERT enzymatic activity and delay replicative senescence in cultured endothelial cells [61,62]. It has been reported that oxidative stress is a regulator of endothelial TERT activity, elevated free radical production and activation of the Src tyrosine kinase. Activated Src then phosphorylates (TERT), which leads to TERT exportation from the nucleus. Undeniably, the age-associated reduction in TERT enzymatic activity can be inhibited by antioxidant and statin interventions, which concomitantly counterbalance free radical formation [63].

Senescent endothelial cells are sensitive to apoptotic stimuli due in part to decreased NO production levels [64]. However, culturing of endothelial cells without any intervention leave these cells vulnerable to apoptosis [65] and the importance of endothelial cell remains unclear. There is no doubt that endothelial cell apoptosis occurs in an in vivo setting. Various stimuli (i.e., pro-inflammatory cytokines, turbulent blood flow) appear to promote this process [66].

The void in the endothelial monolayer after cell injury or programmed cell death/necrosis has to be repopulated. This fulfilment is thought to be brought about via three [3] different mechanisms: (1) expansion and proliferation of neighboring endothelial cells, (2) increase in size of existing endothelial cells and (3) differentiation of endothelial progenitor cells [67,68]. The significance of each of these processes to endothelial cell post-injury regeneration remains unclear and is an intense focus of research. It seems likely that endothelial regeneration may result from a combination of all three mechanisms. There is substantial evidence that aging is associated with a reduced ability of the endothelium to heal and replenish. It is highly unlikely that this is solely a consequence of endothelial aging and other factors such as reduced secretion of and insensitivity to growth factors must also be considered [69]. In the last decade, endothelial renewal via circulating progenitor cells derived from bone marrow has gained particular attention [70]. The number of endothelial progenitor cells (EPCs) decrease with advancing age due to impaired mobilization from the red bone marrow [71]. It has also been reported that EPCs from elderly human subjects have a decreased capacity to engraft. Other studies suggest that the regenerated endothelium is physiologically impaired. This regenerated endothelium is dysfunctional [71], displaying an increased uptake of low-density lipoprotein (LDL) and reduced NO production. In rat models, advancing age appears to impair endothelial regeneration after tissue trauma, which in large part appears to be a consequence of decreased expression and production of eNOS. However, it has been reported that exogenous supplementation of the amino acid arginine, partially restores eNOS levels observed in younger populations which suggest that age-associated depletion may play be involved in this process [72]. The age-associated loss of eNOS activation in the aorta from old rats has been reported to be the result of reduced enzymatic activity of the protein kinase Akt [73].

Angiogenesis declines with advancing age. It has been reported vascular endothelial growth factor (VEGF)-mediated angiogenesis in both aged rats and rabbits [74,75] declines after 12 months of life. It is further reported that angiogenesis-dependent tumorigenesis is inhibited with age [76]. Several reports have described the wound healing process is delayed in aged mice and that this delay is attributed, in part, to an impaired angiogenic process [77,78,79]. As previously mentioned, in connection with age-associated decreased regenerative capacity of the endothelium, endothelial gene expression changes may also have a role in this phenomenon. The inflammation-driven expression of intercellular adhesion molecule 1 (ICAM-1) is reduced in endothelial cells from aged subjects [80]. In both aged mice and in vitro human microvascular endothelial cells, the expression of the extracellular matrix preserving protein metalloproteinase-2 (TIMP-2) is significantly increased. This increased expression correlates with a reduced ability of endothelial cells to degrade and rearrange components of the extracellular matrix, a process necessary for angiogenesis [81]. In mice, inflammation and matrix deposition are also reduced with advancing age. Moreover, the expression of VEGF and transforming growth factor β1 (TGF β1) is reduced with advancing age, while the expression of thromospondin family protein thrombospondin-2 increases. VEGF promotes endothelial proliferation and migration, whereas TGF β1 is involved in matrix formation. Thrombospondin-2, in contrast, inhibits angiogenesis by reducing endothelial cell migration and proliferation [82].

### 1.5. Glucose Tolerance and Aging

The commonality of impaired glucose tolerance (IGT) and diabetes mellitus (DM) increases with advancing age [83], and mechanisms of age-associated glucose intolerance include (1) reduced insulin sensitivity, (2) diminished physical activity (exercise), (3) elevated body fat composition, (4) decreased pancreatic output (β-cell function), and (5) altered dietary practices [84]. Although diabetes in the elderly is often a result of insulin resistance (type 2), the metabolic dysfunction associated with advancing age suggests that elderly persons may differ from younger populations with type 2 diabetes. Even more profound is that non-obese individuals above the age of 60 show a distinct impairment in insulin release coupled with subtle insulin resistance, as opposed to elderly individuals, and have marked insulin resistance even in the presence of adequate levels of insulin in circulation [85]. Drug use, other pathologies and adverse stress all negatively affect glucose in elderly subjects [86].

### 1.6. Hyperglycemia, Diabetes, and Aging

There is an abundance of evidence (pathological and epidemiological) that hyperglycemia or diabetes are a major risk factor for Cardiovascular diseases (CVD) in both men and women. In fact, CVDs are listed as the cause of death in approximately 65% of persons with diabetes [87]. The feedback loop between elevated plasma glucose and diabetes serves as a strong and driving risk factor for several forms of CVD. Even more direr, when patients with diabetes develop clinical CVD, they usually have a worse prognosis for survival than their non-diabetic CVD counterparts [88].

Atherosclerosis is perhaps the biggest danger to the macrovasculature of both patients with and without elevated glucose and diabetes. The etiology and onset of atherosclerosis has been described previously. However, several factors characteristic to high blood sugar and diabetes are worth mention. Dyslipidemia is also strongly correlated with atherosclerosis, and more than two-thirds of diabetic patients are dyslipidemic [89]. Moreover, abnormalities in the physical morphology of the lipoprotein particles have also been reported [90]. In type 2 diabetic patients, the dominant form of LDL cholesterol is the small, dense form; The smaller the LDL particles, the more atherogenic they are. This is in large part because they can infiltrate and firmly adhere to the arterial wall, and because of the smaller surface area, they are more susceptible the detriments of oxidation. In addition, less cholesterol is packaged and transported in the core of small LDL particles compared to that of large particles [91,92]

Oxidized LDL are also pro-atherogenic. This is mediated in part by the immune system. When LDLs are oxidized, they transform and display biomarkers that are recognized by the immune system as foreign. As a consequence, oxidized LDLs produce multiple deleterious biological responses, including drawing leukocytes to the intima of the vessel, reducing the ability of the leukocyte lipid uptake and subsequent differentiation into foam cells [93], of which are critical in the onset of atherosclerotic lesions. In hyperglycemic and diabetic patients, LDL particles can become glycated [94]. Glycation of LDLs extends its circulation ability and therefore increases the ability of the LDL to facilitate the onset of atherogenesis. However, glycation of HDL reduces its half-life and renders it less effective in combating atherosclerosis [95].

Other effects of diabetes on the microvasculature include altered triglyceride dynamics. Diabetic blood is high in triglycerides (hypertriglyceridemia). This is in part due to insulin regulation of lipid fluctuations. Insulin modulates the enzyme lipoprotein lipase, which in turn regulates free fatty acid uptake into fatty tissue. Insulin also reduces the activity of the enzyme lipase through a feedback mechanism, resulting in the attenuated release of free fatty acids into the blood circulation [96,97,98].

The effects of hyperglycemia and diabetes are also characterized in the microvasculature. Aside from canonical association with the eye (retinopathy), kidney (nephropathy), and autonomic function of the nervous system (neuropathy) [99,100,101]. However, small vessels are also affected by elevated blood glucose levels, including those in the peripheral vasculature. Of worthy mention, microvasculature damage and atherosclerosis are mutually exclusive; neither variable is entirely indicative of lipid levels. Unlike the macrovasculature, where atherosclerosis is the major danger, many biomechanisms contribute to microvascular disease as a consequence of elevated blood glucose and diabetes.

Microcirculation is regulated by two mechanisms: central regulation (nervous system) and local regulation (endothelium). The former is mediated by sympathetic and parasympathetic nerves stimulate VSMCs. Local regulation is mediated via vasodilator (including NO) and vasoconstrictors (endothelin-1) produced via the endothelial cells. Under normal physiological conditions, the vascular smooth muscle receives constant nerve signals and a steady supply of nitric oxide (NO) from the endothelium. These mechanisms balance microvascular flow quickly to meet the metabolic demands of the tissue [102,103,104,105].

Elevated blood glucose and diabetes confer dysfunction in both the autonomic nervous system and the endothelium, which often result in microvascular complications. Diabetic autonomic neuropathy (DAN) is one common clinical occurrence associated with dyfunctional blood flow in various vascular beds, including those located in the heart [99,100]. Patients suffering with DAN have higher rates of sudden cardiac death and cardiovascular mortality rate. Such patients are reported to have an abnormal cardiac flow reserve that is activated in response to cardiac stress [106].

Patients with hyperglycemia and diabetes have been reported to have attenuated bioavailability of NO, as described previously, as well as reduced secretion of endothelin-1. Vasoconstriction has been found in subjects with diabetes [107,108]. Elevated glucose and diabetes attenuate NO availability in part to insulin deficiency and/or insulin resistance in endothelial cells [104]. Further, elevated blood glucose levels periodically inhibit the production of nitric oxide in arterial endothelial cells [108].

The primary function of blood flow and circulation is the transport and exchange of substances between blood and tissue fluid. Regardless of adequate blood flow, processes that halt gas and nutrient exchange will disrupt the homeostasis of the tissue associated with corresponding vascular bed. Capillary basement membrane enlargement associated with prolonged hyperglycemia is also structural hallmark of diabetic microvascular disease. This enlargement negatively impacts the amount of transport of metabolites and nutrients between the circulation and the tissue. This may be partially responsible for the poor exercise tolerance observed in subjects with the diseases.

Delivery of substances from the blood circulation into tissue interstitial space is mediated by many mechanisms. These include, but are not limited to, (1) blood pressure, (2) systemic blood flow, (3) substance size and (4) charge specificity. Counterintuitively, enlargement of the basement membrane increases microvascular permeability [93]. This increased porosity allows for the infiltration of large molecules normally excluded from passage across the vasculature. Clinically speaking, trans-capillary leak of proteins in the kidney provides an important indicator of microvascular disease [107].

### 1.7. Hypoglycemia and Aging

Hypoglycemia is defined by an approximate plasma glucose level of 54–70 mg/dL. Within the clinical community, however, the threshold and precise definition of clinical hypoglycemia remains unclear. The severity of hypoglycemia can be defined as either “self-treatable” in which the individual is asymptomatic or “biochemical hypoglycemia”, an state that requires medical intervention for recovery [108].

Hypoglycemia is common in both diabetics and non-diabetics due in large part to clinical attempts achieve tight blood glucose regulation, although this has been shown to be harmful to the overall health of the individual [109]. Few and limited studies have examined the frequency and impact of hypoglycemia in non-diabetic patients with not much more reported in the elderly (compared to the extensive literature on hyperglycemia and diabetes). While hypoglycemia in older people, especially those with diabetes is common, its recognition can sometimes be arduous. Further, the clinical manifestations of acute mild hypoglycemia are often non-or misdiagnosed. For example, hypoglycemia in elderly patients with diabetes often manifest symptoms such as dizziness or visual disturbances, which are misdiagnosed [110]. Diabetes in older patients can also be mistaken for dementia, which increases with advancing age. Both dementia and hypoglycemia entail symptoms of agitation, confusion or behavioral abnormalities. Symptoms of hypoglycemia tend to be less specific with advancing age. In one such report on a study of hypoglycemia symptoms, more than 50% of patients (older people with diabetes) referenced non-specific symptoms of “general unwellness” when their blood glucose level decreases, which may make the identification of hypoglycemic episodes more difficult for clinical specialists [111]. In the elderly, the threshold of symptoms of hypoglycemia occurs at lower blood glucose level and cognitive dysfunction occurs at a higher level compared with younger adults; hence, both autonomic and neurological symptoms occur almost at the same time with little or no warning (impaired awareness of hypoglycemia). Mild hypoglycemia or episodes with few or no symptoms may further attenuate awareness of a severe episode because the glycemic threshold has been previously lowered. Stated another way, mild episodes of hypoglycemia can induce further episodes of more severe hypoglycemia [112].

Glucose is the primary metabolic fuel for the brain under normal physiological conditions. The brain cannot synthesize glucose de novo. As a result, sustainment of brain function requires an abundant and continuous supply of glucose from systemic circulation. In normal situations, glucose counter-regulatory mechanisms (i.e., hormones, buffering) efficiently prevent or rapidly counterbalance hypoglycemic episodes [113].

When hypoglycemia occurs, a hormonal response is induced by way of insulin secretion. In addition to insulin secretion, other hormones such as glucagon and adrenalin are also secreted in prompt response to falling plasma glucose levels. These hormones, along with insulin, both cause an increase in glucose production [114,115].

The numerous episodes of hypoglycemia further attenuate the sympatho-adrenal glycemic threshold to a lower plasma glucose levels; this is most notable in patients with an intensive regulation of diabetes. The collective combination of glucagon inhibition and weakened adrenalin response causes defective glucose counter-regulation, a clinical manifestation that has been shown to augment the prevalence of severe hypoglycemia by greater than 20-fold (or even higher) during therapeutic intervention compared to when a normal adrenalin is presented [116]. Decreased systemic response to adrenalin release is a marker of an “attenuated autonomic neural response”. This causes, in part, clinical syndrome of hypoglycemia unawareness, a condition that contributes to the onset and progression of severe hypoglycemia development. Hypoglycemia-associated autonomic failure (HAAF) in type 1 diabetes appears to be the consequence previous episodes of hypoglycemia that have been caused by defective counter-regulatory response and hypoglycemia unawareness. It is then plausible to link HHAF-induced hypoglycemia to a role in the vicious cycle of recurrent hypoglycemia.

The causes and risk factor(s) for hypoglycemia remain unclear, but in general, manifestations occur rarely under normal physiological conditions, unless sustained fasting for various reasons. However, hypoglycemic episodes occur in diabetic patients more frequently, with an estimated 90% of all diabetic experiencing at least one hypoglycemic episode. Hypoglycemia occurs when the homeostatic balance between insulin intake and body’s physiological need is disrupted. In addition to iatrogenic and fasting-induced hypoglycemia, hypoglycemia has also been reported to stem from attenuated immune function and the ability of the body fight off infection with advancing age [11,117].

Diabetes medications (i.e., insulin, and sulphonylureas) are among the leading causes of hypoglycemia in diabetic patients. Metabolically chronic sulphonylureas have been linked to more severe episodes of hypoglycemia than transient compounds [118]. Hypoglycemia with metformin have also been reported when an imbalance between food consumption and dose of metformin are consumed [119].

Hypoglycemia may also result from scarce foods consumption or increase in activity in relation to medicinal and food intake. Alcohol consumption (via gluconeogenesis inhibition), drug use, various stress, and infections should also be considered in the contribution of hypoglycemia in diabetic subjects

Low blood sugar could also be a symptom of advanced organ dysfunction. Many liver and kidney diseases have been implicated diseases have been associated with induction of hypoglycemia. Not surprisingly, these two organs have a major role in glucose production and maintenance of blood sugar levels.

In type 2 diabetic patients, both insulin deprivation and duration of insulin treatment increase the risk of hypoglycemia. The risk of a hypoglycemic episode is highest in those patients with type 2 diabetes and who have been insulin treated for more than 10 years [120]. The risk of severe hypoglycemia is higher in with advancing age and those with co-morbidities. [121].

The effects of hypoglycemia not only affect neurological function, but have cardiovascular implications as well. Several reports have highlighted a link between iatrogenic-induced hypoglycemia and acute vascular events (i.e., angina; [122]). Other studies have described an increased risk of mortality and microvascular dysfunction when glucose levels are tightly regulated. However, these findings are not without some controversy. The Action to Control Cardiovascular Risk in Diabetes (ACCORD) trial not could definitively establish a clear association of hypoglycemia in increased cardiovascular events [123]. However, a more compelling link between severe hypoglycemia and vascular events is indicated in the Veteran’s Affairs Diabetes Trial (VADT) study [124]. One such study suggested that abundant onset of hypoglycemia may initiate and propagate the development of macrovascular disease in type 1 diabetes. This occurs indirectly by increasing the risk of atherosclerosis. Sympathetic release of hormones during acute hypoglycemia causes cardiovascular changes including leukocyte activation and release. Altered blood flow and localized vasoconstriction together with a damaged endothelium, in part, promote tissue ischemia. Extensive hyperglycemia likely drives pre-clinical atherosclerosis in diabetic patients. Recurring episodes of hypoglycemia may contribute to this process by intensifying and accelerating current micro and macrovascular complications [125].

There may be a link between severe hypoglycemia and cognitive impairment. Premature exposure to severe hypoglycemia could affect cognitive abilities many years later. In one report, cognitive scores of ten diabetic children who experienced severe hypoglycemia before age of ten were statistically less than the eighteen diabetic children without a history of severe hypoglycemia. This correlation was consistent 16 years later when all subjects were re-examined [126].

Another study found that severe hypoglycemia appears to be correlated with extensive brain damage in cortex and the hippocampus. Interestingly, the extent of damage mimicked symptoms consistent with seizure-like activity. This finding suggests heightened sensitivity of the cortex and hippocampus following an episode of hypoglycemia [127].

### 1.8. Serum Response Factor (SRF)

Serum response factor (SRF) is an original member of the MADS (Mcm1, Arg80, Agamous and Deficiens, and SRF) domain-containing family of transcription factors and is expressed in most species in the animal kingdom. Its expression increases with advancing age. SRF is named due its binding and high affinity to a serum response element (SRE) within the promoter region of the transcription factor, fos [128]. Other SREs have also been found in the promoter regions of a number of other immediate-early transcription factors. Regulatory signaling in response to SRF occurs mostly via the mitogen-activated protein kinase (MAPK) or RhoA GTPase pathways that converge to induce gene expression [129]. SRF-related genes encode for proteins that initiate and modulate growth signaling factors and cell cycle genes [130]. Hence, SRF appears to be a master driver of cell growth and proliferation. In vitro support for this comes from functional studies that analyzed gain- and loss-of-function assays. The SRF transcription factor’s role as a growth-associated DNA-binding factor is demonstrated substantially, there are also reports of SRF-associated cell growth repression. In one study, it has demonstrated that SRF alters the ras-transformed phenotype [131]. Other in vitro evidence antagonizing the notion of SRF being mandatory for cell proliferation includes studies on SRF-null embryonic stem cells. In these studies, while impaired expression of SRF target genes are clear and convincing, cells still proliferate normally [132]. This seems to support the possibility that other signaling pathways exist in cells to ensure that growth occurs, even in the absence and/or deactivation of the serum response factor. One thing is quite clear: in cells where SRF expression is attenuated, there is no cytoskeletal-contractile function.

Damage to the vessel wall induces the rapid increased expression of the immediate-early c-fos transcription factor in addition to other SRF-regulated genes involved in growth and proliferation of VSMCs and endothelial cells [133]. SRF can also regulate repair and regeneration in epithelial cells in in vivo models of peptic ulcers [134,135,136]. In the same such model, anti-sense mediated knock-down of SRF delays angiogenesis. In yeast models, mutations of the mini-chromosome maintenance 1 (Mcm1) gene results in defective DNA replication [137]. In contrast to the aforementioned findings, much in vivo genetic evidence also exists for SRF playing a pivotal role in processes separate from cell proliferation. One study demonstrated cardiac-specific overexpression of either wild-type or dominant-negative SRF results in various forms of heart failure due to significant changes in contractile gene expression [138]. It has been further demonstrated in multiple models that SRF inactivation. or knockdown, does not lead to impaired proliferative ability, but rather highlights a necessary and primordial role for SRF in the organization and maintenance of the cytoskeleton. It is again clear to reiterate that while the data seem to demonstrate that SRF is not essential for cell division and tissue growth, when it is present, it likely plays a role in growth-associated processes.

Further studies in the bioenergetics field showed SRF-SRE binding in the promoter of over 25 contractile genes that mark the mature phenotype of sarcomeric muscle [139]. These studies were the first of many that have demonstrated that SRF can regulate many contractile genes. Further developmental analysis demonstrate the highest levels of SRF gene expression in differentiating cardiac, skeletal, and smooth muscle cell lineages. The finding that SRF regulates incompatible pathways of gene expression (growth vs. muscle differentiation) sparked interest in both its mechanism and binding promiscuity. Through its nearly 60 binding cofactors, SRF mediate its transcriptional regulatory effects to cell-specific gene clusters.

Among the various SRF cofactors, myocardin (MYOCD) has been of interest as it pertains to the vasculature, moreover in VSMC differentiation [140]. MYOCD is one of the most powerful and essential transactivators known in eukaryotic systems. Through binding with SRF, MYOCD can coordinate a near-complete program of smooth muscle cell differentiation [141,142,143,144,145]. Interestingly, MYOCD knockout studies in mice reveal a quick mortality shortly after formation of a functional cardiovascular system due to a defective vascular SMC differentiation. Two other myocardin-related transcription factors (MRTF-A and MRTF-B) appear to also be important and are expressed more than MYOCD [146]. These isoforms drive distinct patterns of gene expression.

Many SRF target genes have distinct physiological functions via the actin cytoskeleton and contractile systems. However, it should be noted the number of actin cytoskeleton genes regulated by SRF vastly surpasses those of contractile genes [147]. SRF-associated genes are known to be regulated by dynamic alterations in the microfilament cytoskeleton. The RhoA protein, which drives the synthesis of stress fibers, can mediate SRF-associated gene expression [145,148]. Later studies show that increases in SRF activity via RhoA signaling was through the reduction in globular (G) actin during simultaneous filamentous (F) actin formation [145]. Two well-studied RhoA-dependent pathways appear to mediate a reduction in G-actin levels: the formin family of proteins and the ROCK-cofilin pathway. Both cascades work to balance F- to G-actin. One result of attenuating G-actin levels is through the isolation of MRTF-A. MRTF-A is normally bound to G-actin in the cytosol. Once released from G-actin, MRTF-A translocate inside the nucleus where it associates with SRF to regulate gene expression [146]. SRF also has the ability to translocate between the nucleus, the cytoplasm, and cell membrane. β-actin is found in the nucleus and is believed to be involved in the assembly of a specific pre-initiation complex for transcription [149]. Moreover, many actin cytoskeleton genes are themselves direct targets of SRF via an SRE regulatory element, suggesting the concept of a feedback loop for SRF-dependent direct control of the actin cytoskeleton.

**Figure 3:**
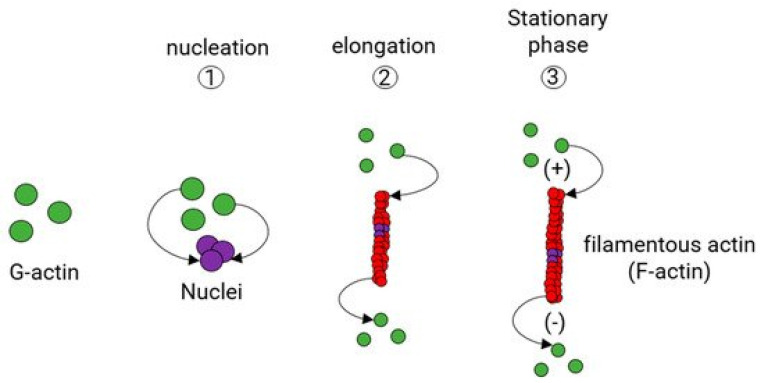
Actin treadmilling mechanism. Actin elongation and breakdown are contingent upon ATP levels; in f-actin elongation, g-monomers are “charged” (ATP rather than ADP + Pi) and are added to the positive (+) end. As it treadmills down towards the positive end, ATP is hydrolyzed to ADP + Pi, becomes unstable, and susceptible to cleavage proteins.

Cytoskeletal actin dynamics, long believed to be constant anchor responsible for the maintenance of cell shape and polarity, is subject to changes involving a plethora of proteins that in part facilitate the cytoskeleton as a whole [150,151]. One of the keystones of actin filament dynamics and cellular motility is a process known as “actin treadmilling.” Actin treadmilling is essential in RhoA-mediated SRF activation (Figure 3; [152]). 

Many SRF target genes encode for proteins that are essential in actin treadmilling. One protein, profilin 1 (PFN1) is reported to tether G-actin, but can readily coordinate the polymerization of G-actin into F-actin via its ability to exchange ADP exchange for ATP-actin; the latter is the more polymerization form of actin (charged). In addition to actin-forming genes, four well-known actin depolymerization proteins (destrin, cofilin-1, cofilin-2, and gelsolin) have been shown to be direct targets of SRF. In particular, gelsolin destabilizes F-actin filaments in a calcium-dependent manner. Cells lacking gelsolin have elevated stress fibers and lacks in ability to migrate. Destrin and cofilin reduces F-actin by promoting the dissociation of ADP-actin (uncharged) at the pointed (positive) end of actin filaments, thereby increasing pools of ADP-actin for sequestration by formin. Destrin and cofilin-mediated f-actin breakdown is relatively balanced, in part, through competitive binding of pro-F-actin stabilizing proteins such as tropomyosin. It is important to highlight that both destrin and cofilin activity is negatively regulated by kinase activity; this phosphorylation occur through RhoA-mediated signaling, which ultimately activates SRF [150], as previously stated. The fate of phosphorylated destrin-cofilin is unclear, but it is intriguing to consider that such proteins could be destined for degradation or upregulation.

## 2. The Aging Cytoskeleton: Glucose Sensitivity

It was long held the cellular cytoskeleton was a tethering platform to which most organelles were associated. However, in the last fifteen years, the cytoskeleton has been implicated in programmed cell death, mitochondrial-to-nuclear signaling, and regulation of metabolism [153,154,155]. Moreover, the actin cytoskeleton has been shown to mediate many cellular processes through rearrangement and fluctuation from monomeric globular actin (g-actin) to polymeric filamentous actin (f-actin). Aging alters the of the actin cytoskeleton by altering the expression of actin [156]. Such changes have been reported analyzing the β-actin expression in rats over a period of 24 months. Compared to the gene expression levels in younger rat populations (six month), reduced levels of actin were observed in 2-year-old rats by nearly 40%. A complimentary finding of the variation in the level of β-actin expression has been reported in crustacean aging models [157,158,159,160]. It is plausible that age-associated fluctuation in microfilament expression is the consequence of age-associated hormonal flux. Actin dynamics are impacted by many hormones including hydrocortisone, and triiodothyronine. Most of the aforementioned hormones secretion frequency is altered with advancing age. For instance, insulin signaling dysfunction is increased in populations aged 65 and older [161].

In correlation with insulin’s effect on actin dynamics, it is intuitive to highlight glucose’s effect on actin dynamics. Actin “treadmilling” or the dynamic transition of (g-actin)-to-(f-actin) is dependent on ATP production; as f-actin is elongated, ATP is hydrolyzed and the f-actin become structurally unstable and susceptible to cleavage by cofilin proteins.

As plasma glucose levels fall, as with hypoglycemia, it is plausible that, in fact, actin dynamics are altered [162]. A recent study showed altered f-actin redistribution is response to in vitro acute glucose, a process driven by molecular co-chaperone protein, p49/STRAP in C2C12 cells [163]. In human umbilical vein endothelial cells (HUVECs), both acute and chronic exposure to hypoglycemic cell culture media accelerated the premature biomarkers of cellular senescence including SA-β-galactosidase staining, increased expression of the cell cycle inhibitor p16^INK4a^, and an altered squamous-like shape; the latter of which was shown to reduce f-actin microfilament expression while increasing the intermediate filament vimentin [164].

## 3. SRF: Glucose Sensitivity

SRF has been implicated in glycemic fluctuation, most notably during in vitro exposure to hyperglycemic conditions. In one study, SRF expression was increased in both human and murine myoblasts in response to elevated glucose, a phenomenon that involved the increased expression of SRF and its coactivator megakaryoblastic leukemia 1 (MKL1). Elevated SRF expression correlated with actin polymerization compared to cells exposed to normoglycemic conditions. In addition to being an SRF co-activator MLK1, like SRF, is a regulator of actin genes. Elevated SRF levels correlated with decreased g-actin monomers, prompting the question of whether SRF, MLK1, or both proteins were needed for the elongation of actin fibers. When SRF was pharmacologically inhibited in the presence of elevated glucose, but not MLK1, actin depolymerization increased, suggesting that SRF is a signature of both elevated glucose and actin polymerization [165].

Prediabetes is a physiological condition where plasma glucose levels are higher than normal, but not high for a type 2 diabetes diagnosis. The reversible transition from “pre-diabetic” to “type 2 diabetes” is driven in part by factors linked to physical activity, habitual practices (i.e., smoking), insulin resistance, and advancing age. In addition, this transition is also linked to nutrition. Given SRF’s expression in response to elevated glucose and advancing age, it is plausible that the prediabetes–diabetes transition is driven in part by the transcription factor. A reported identified an SRF pathway that involves the striated muscle activator of Rho signaling (*STARS*). As insulin resistance progresses, the cytoplasmic STARS protein migrates intracellularly into the nucleus, where it promotes the increased expression of the SRF co-activator MKL1. Through increased MKL1, SRF expression and activation permit the upregulated transcriptional activity of SRF and ultimately the repression of GLUT4. The cellular uptake of glucose is attenuated and plasma glucose remains above normoglycemic levels [165]. This prolonged elevated plasma glucose leads to prediabetes and ultimately type 2 diabetes.

SRF’s sensitivity to elevated glucose is also highlighted in the molecular transition of fatty liver disease to fibrosis. Non-alcoholic fatty liver disease (NFLD) is a condition where liver tissue is replaced by more than 4% adipose tissue. NFLD is characterized by the increased liver fat deposition, which can cause scarring—a condition known as steatohepatitis. Steatohepatitis can lead to liver fibrosis which causes a buildup of scar tissue in the liver. *Progression of steatohepatitis involves the* activation of hepatic stellate cells (HSCs). A key driver in the activation of HSCs appears to be reactive oxygen and nitrogen species (ROS/RNS. Two activation domains of the nicotinamide adenine dinucleotide phosphate (NAPDH) oxidase (NOX) complex, NCF1and NCF2, are upregulated during HSC activation augments ROS/RNS production and ultimately liver fibrosis. In one study, it is reported that HSC-conditional SRF knockout mice demonstrated a similar phenotype of liver fibrosis compared to wild-type mice fed identical diets. Interestingly, SRF knockdown reduced free radical levels in HSCs in vivo. Comparably, SRF repression in cultured HSCs suppressed ROS/RNS production. Further investigation revealed that reduced SRF expression resulted in repression of NCF1/NCF2 expression. SRF-modulated epigenetic activation of NCF1/NCF2 by interacting with the histone acetyltransferase KAT8. This study suggest that SRF integrates transcriptional activation of NCF1/NCF2 and ROS production to promote liver fibrosis [166].

In vitro analysis of C2C12 cells exposed to glucose also implicates a potential role of SRF. In one study, murine myoblasts were subjected to both a chronic and acute low-glucose exposure. During a 6 h time course, it was shown that that SRF gene expression increased during the first continuous hour of glucose deprivation, after which its expression was decreased to comparable levels of those myoblast exposed to normoglycemic conditions [164]. Of importance was the expression of an SRF-binding protein, the co-chaperone p49/STRAP (SRFBP1a). In response to the same low-glucose exposure experiment, p49/STRAP gene and expression levels appeared to inversely correlate with levels observed in SRF expression. After 6 h of low-glucose stress, not only did p49/STRAP gene and protein expression increase over 200%, but diffused out of the nucleolus, into the nucleus and into the cytoplasm. At longer continuous glucose deprivation, p49/STRAP localized to the cellular periphery at the ends of f-actin stained with phalloidin. Interestingly, as p49/STRAP expression increased, a significant amount of f-actin distribution was observed. siRNA-mediated knockdown of p49/STRAP in the presence of low glucose did not appear to preserve f-actin levels, but were significantly higher in non-siRNA treated C2C12 cells exposed to low glucose. In the analysis of these findings, it is quite plausible that f-actin redistribution could be a consequence of reduced ATP levels, but given the SRF-p49/STRAP binding interaction, the correlative expression patterns, their cellular localization and expression during both high- and low-glucose exposures, and the comparative analysis of reduced p49/STRAP reduced on f-actin levels in the presence of low glucose, it is plausible that SRF has a role, either direct or indirect, in the modulation of f-actin during glucose deprivation [163].

## 4. Conclusions

The physiology of aging is driven in large part by nutrition. Elevated plasma glucose levels and the correlation with correlation insulin resistance are well documented in the literature. Moreover, signaling cascades implicated in models of insulin resistance and diabetes are known and involve crosstalk from several different pathways. However, the molecular mechanisms associated with hypoglycemia remain unclear. Here, we report the clinical and the molecular mechanisms known about hypoglycemia and chronic low plasma glucose levels. In addition, we discuss the potential role(s) the serum response factor (SRF) has in in vitro studies in mechanisms observed during glucose deprivation. Cytoskeletal rearrangements observed in cellular aging models may in part be driven by SRF expression through microfilament rearrangements. Moreover, changes in cell growth and population doubling levels may also be both directly and indirectly regulated by SRF at the genetic level. The promiscuity of SRF to other downstream kinases and transcription factors leads to the speculation that SRF is not the “master regulator” of the glucose fluctuation cellular changes, but may be a key driver of the cellular glucose stress response. Future studies investigating the SRF protein–protein interaction under normoglycemic vs. hypoglycemic conditions will be useful in further elucidating the molecular pathways involved in the hypoglycemic stress response.
